# Knowledge, awareness, and use of folic acid among women of childbearing age living in a peri-urban community in Ghana: a cross-sectional survey

**DOI:** 10.1186/s12884-024-06408-z

**Published:** 2024-04-05

**Authors:** Obed Akwaa Harrison, Idolo Ifie, Chikere Nkwonta, Bennett Atta Dzandu, Albert Owusu Gattor, Emma Efua Adimado, Kofi Kafui Odoi, Benedicta Aziavor, Firibu Kwesi Saalia, Matilda Steiner-Asiedu

**Affiliations:** 1 Department of Nutrition and Food Science, University of Ghana, Legon, Accra Ghana; 2 Department of Food Science, University of Leeds, Leeds, England; 3 Faculty of Chemistry and Pharmacy, Medicinal Chemistry II, University of Regensburg, Regensburg, Germany; 4 Institutional Care Division, Ghana Health Services, Accra, Ghana; 5 Department of Education, University of Ghana, Legon, Accra Ghana; 6 Department of Food Process Engineering, University of Ghana, Legon, Accra Ghana

**Keywords:** Folic acid, Folates, Vitamin B_9_, Awareness, Knowledge, Neural tube defects

## Abstract

**Background:**

Folic acid, a water-soluble B-complex vitamin, plays a crucial role in DNA synthesis and maintenance, making it particularly significant during reproduction. Its well-known ability to reduce the risk of congenital anomalies during the periconceptional period underscores its importance. The increased requirement for folate during pregnancy and lactation is essential to support the physiological changes of the mother and ensure optimal growth and development of the foetus and offspring. This study assessed the knowledge, awareness, and use of folic acid among pregnant and lactating women of reproductive age residing in Dodowa in the Shai Osu-Doku District, Accra, Ghana.

**Methods:**

The study was a cross-sectional design that involved 388 randomly selected participants (97 pregnant and 291 lactating women). Structured questionnaires were administered to gather information on the socioeconomic demographic characteristics, knowledge, awareness, and use of folic acid of the participants. Dietary intake was assessed using a food frequency questionnaire. The data were analysed using descriptive statistics and Pearson’s chi-square analysis tests and are presented as frequencies and percentages, means, standard deviations, bar graphs, and pie charts. The significance of the results was determined at a 95% confidence interval.

**Results:**

The mean age of the participants was 31 ± 5.0 years. Among the study participants, 46.1% demonstrated knowledge of folic acid deficiency, while approximately 68.3% had a high awareness of folic acid supplementation. Approximately 75% of the participants indicated that they had not used folic acid supplements within the week, and 15.5% reported consuming folic acid-fortified food per week.

**Conclusions:**

The women exhibited high awareness but poor knowledge regarding the usage of folic acid supplementation during pregnancy and lactation. Consequently, this lack of knowledge influenced the low use of folic acid supplements and low intake of folate-rich foods among pregnant and lactating mothers.

## Background

Folate has been extensively studied and demonstrated to have a crucial role in reducing the risk of neural tube defects (NTDs) in foetuses and megaloblastic anaemia in women of reproductive ages, as well as their offspring [1]. Estimates by Roger et al. [2] suggest that worldwide, approximately 500,000 children are born with spina bifida and anencephaly each year, and fortifying staple foods with synthetic folic acid has great potential to prevent folate-preventable neural tube defects, but currently, only approximately a quarter of that fortification potential is being averted [3]. The aetiology of neural tube abnormalities is multifaceted, involving a combination of genetic and environmental factors, including nutrition. Extensive intervention studies conducted globally have shown that even a relatively small dose of folic acid supplementation can protect against neural tube defects, and it is widely acknowledged that maternal intake of sufficient folic acid before and during early pregnancy (0.4 mg per day) can prevent between 50% and 70% of affected births [1].

In Africa, the estimated prevalence of neural tube defects is 1–3 per 1000 births annually [4, 5]. Although there are no official national statistics on the prevalence of NTDs in Ghana, some studies have reported estimates ranging from 1.15 to 1.6 per 1000 live births and stillbirths, varying depending on the study [4, 6]. Moreover, there is evidence suggesting that the low awareness of NTDs in Africa has hindered efforts to combat these defects [7]. Marrow samples have indicated that megaloblastic anaemia is found in 24–60% of pregnant and lactating women who do not regularly take prenatal folate or cobalamin supplements [8]. According to the Ghana Micronutrient Survey 2017 [9], the prevalence of folate deficiency among nonpregnant women aged 15 to 49 years in Ghana is estimated to be 53.8%, irrespective of their educational attainment, household wealth, or urban/rural residence. These findings emphasize the public health significance of folate deficiency among Ghanaian women, which can be attributed to factors such as lack of knowledge and low utilization of folate.

Therefore, it is imperative to explore the knowledge and use of folate among women of reproductive age, as it represents a significant barrier to preventing birth defects and promoting folate consumption either as a supplement or from foods.

## Methods

### Study design and setting

This study was a descriptive cross-sectional survey conducted in Dodowa, located in the Shai Osu-Doku District, Ghana. The Shai Osu-doku District is one of the ten districts comprising Ghana’s Greater Accra Region. Geographically, it is situated in the southeastern part of Ghana, spanning latitudes 5° 45′ south to 6° 05′ north and longitudes 0° 05′ east to 0° 20′ west. The district encompasses a land area of 1528.9 square kilometres, accounting for approximately 41.5% of the entire region. Dodowa serves as the administrative capital of the district. According to the Ghana Statistical Service, the district’s projected population is 67,105 individuals, with females representing 51.3% (34,425) and males comprising 48.7% (32,680) of the total population [10]. Notably, the district is predominantly rural, with approximately 76.7% of the population residing in rural areas, while only 23.3% reside in urban and peri-urban areas.

Some of the communities within the district are rapidly urbanizing due to their proximity to Accra, the capital of Ghana. The district is surrounded to the northeast by North Tongu District to the northeast, Yilo Krobo Municipality and Upper Manya District to the northwest, Akwapim North Municipality to the west, Kpone Katamanso Municipality to the southwest, Ningo-Prampram District to the south, and Ada West District to the east. Agriculture (farming, fishing) and small trading, which employ 58.6% of the population, are the district’s mainstays [10].

### Sample size and study population

The sample size was calculated using the formula below, developed by [11]. The sample size was calculated using a 53.8% prevalence of folic acid deficiency among women aged 15 to 49 [9].

$${\text{N}}=\frac{{{{\text{Z}}^2} \times {\text{ P }}\left( {1 - {\text{P}}} \right)}}{{{{\text{D}}^2}}}$$

where:

N = the minimum required sample size.

Z = the critical value for the 95% confidence level (1.96).

P = the estimated prevalence of folic acid deficiency in Ghana (0.538).

D = the margin of error (0.05).

$$\begin{aligned} {\text{N}} & =\frac{{{{1.96}^2} \times 0.538\left( {1 - 0.538} \right)}}{{{{0.05}^2}}} \\ {\text{N}} & =381.94 \approx 382 \\ \end{aligned}$$

5% was added to the predicted sample size to account for the risk of nonresponse or incomplete questionnaires. As a result, the study’s overall sample size was estimated to be 400 pregnant and lactating women of reproductive ages.

Sampling method.

In this study, simple random sampling using the modified random walk door-to-door approach described by Flynn et al. [12] was employed to select a total of 388 pregnant and lactating women of reproductive age, between 15 and 49 years, in Dodowa. The modified random walk door-to-door approach integrates a stochastic element into traditional canvassing or data collection strategies. By selectively targeting doors in a semi-random manner, informed by algorithms that consider various factors like demographics, prior engagement, and geographic specifics, this method aims to minimize selection bias and enhance the diversity of the collected data or the outreach impact. The approach strikes a balance between randomness and strategic selection, ensuring a wider and more varied interaction base. This not only enriches the quality and representativeness of the data or feedback gathered but also potentially uncovers new insights by reaching previously overlooked segments [12]. The selection of study participants was based on predefined inclusion and exclusion criteria. The inclusion criteria were pregnant and lactating women aged between 15 and 49 years who possessed effective communication skills and demonstrated a willingness to participate in the study. Potential participants who exhibited apparent health issues and non-residents of the study area were excluded.

### Data quality assurance

The questionnaire was pretested at Sota, which is a neighbouring community. The pregnant and lactating women in this town had similar characteristics as the pregnant and lactating women in Dodowa. Based on the responses collected, suitable changes to the questionnaire were made to ensure reproducibility, clarity, and accuracy. The raw data received from the survey were kept on the Kobo Collect data management system, and data were collected with tablets and mobile devices, with only the researcher and supervisors having access to it. The respondents’ anonymity was guaranteed by issuing codes. To maintain anonymity and confidentiality, names and personal descriptive data were also removed.

### Survey instruments and interviewing

A semi-structured questionnaire was used to conduct the survey. The questionnaire had four sections, three of which were drawn from the study’s goal: knowledge as well as awareness and use of folic acid and frequency of consumption of naturally occurring folate, while the other was background information. The research instrument had 21 questions. The survey was designed in English; however, it was administered in both English and the local dialect (Twi, Fante, Ga, or Ewe). Interviews were conducted in the households of the participants by trained field assistants who were multilingual. This allowed pregnant and lactating women who could not speak English to comprehend and appropriately and comfortably respond to the questionnaire. The questionnaires were constructed based on relevant literature and recommendations from experts.

### Data processing and analyses

Questionnaire information was coded, entered, and analysed using the Statistical Package for Social Sciences (SPSS) version 27.0 for Windows and reported using tables and figures. Descriptive statistics of frequencies, percentages, means, and standard deviations were used to describe the characteristics of the pregnant and lactating mothers: age, education level, marital status, ownership of items, sources of food, lighting, and water, Folic acid/folate knowledge, Folic acid/folate use and attitude towards Folic acid/folate usage. Folic acid/folate knowledge and awareness were determined based on nutrition knowledge scores. Scores were coded as 1 for a correct response and 0 for an incorrect response. The overall Folic acid/folate knowledge and awareness score for women was determined by the number of accurate responses; 6 questions yielded a total score of 6 as such, a score of 4–6 was classified as good knowledge and awareness and those less than 4 were classified as poor folic acid/folate knowledge and awareness. In this study, Knowledge of folic acid/folate was defined by a set of questions on the food sources of folic acid, the importance, consequences of deficiency on pregnancy and childbirth as well as the occurrence of neural tube defects. Awareness of folic acid/ folate covered issues such as source of information on folic acid and general consciousness and use of folic acid/folate supplements. Pearson’s chi-square analysis was performed to establish relationships between Folic acid/folate knowledge scores, awareness scores, and the use of folic acid supplements. The significance of the results was determined at a 95% confidence interval.

## Results

Table 1 profiles the background characteristics of the participants. A total of 388 participants comprising of pregnant (25%) and lactating (75%) women actively participated in the study, and the Ga-Adangbe ethnic group was the dominant group (38.4%). The mean age of the study participants was 31 ± 5 years. Most women (72.9%) fell within the 30–39 age group. In terms of educational attainment, approximately one-third of the respondents (36.3%), had completed junior high school, and the lowest, 3.6%, had no formal education. Christianity was the predominant religion among the respondents, accounting for 93.6% of the participants, and the rest fell within other religious groups (Muslims, Judaism, Hinduism, and Buddhism). More than half of the participants (53.9%) reported being unemployed. Concerning marital status, approximately 71.9% of the respondents were married, while the remaining 18.3% were unmarried, and 9.8% were cohabiting.

**Table 1:** Sociodemographic characteristics and birth history of participants (*N* = 388)

Respondents’ characteristics	n (%)
**Reproductive status**	
Pregnant	97 (25.0)
Lactating	291 (75.0)
Age (years) (M ± SD)*	31 ± 5.0
15–19	18 (14.6)
20–29	84 (21.6)
30–39	283 (72.9)
40–49	3 (0.8)
**Religion**	
Christian	363 (93.6)
Muslim	19 (4.9)
Other^a^	6 (1.5)
**Ethnic group**	
Akan	117 (30.2)
Ewe	100 (25.8)
Ga-Adangbe	149 (38.4)
Mole-Dagbani	10 (2.6)
Other^b^	12 (3.1)
**Educational level**	
No education	14 (3.6)
Primary	48 (12.4)
Junior High School	141 (36.3)
High School Education	25 (6.4)
Vocational	102 (26.3)
Tertiary	58 (14.9)
**Occupation**	
Employed	179 (46.1)
Not employed	209 (53.9)
**Monthly income (GHS)**	
399 or less	140 (36.1)
400–1000	25 (6.4)
1001–1999	3 (0.8)
2000–2999	55 (14.2)
**Marital status**	
Not married	71 (18.3)
Cohabiting	38 (9.8)
Married	279 (71.9)
**Planned pregnancies**	
Yes	71 (18.3)
No	308 (79.4)
Not applicable (for women without children)	9 (2.3)
**Gravidity**	
0–2	102 (26.3)
3–4	278 (71.6)
4 or more	8 (2.1)
**Parity**	
0–2	219 (56.4)
3–4	169 (43.6)

Other^a^; refers to Judaism, Hinduism, and Buddhism. Other^b^; refers to krobo and Fulani, * Mean ± Standard Deviation

Regarding monthly income, it ranged between Ghs 399 or less to 2999, with 36.1% of the participants having a monthly income of Ghs 399 or below, while only 14.2% reported a monthly income between Ghs 2000–2999. Notably, a significant proportion (80.2%) of the participants reported having unplanned pregnancies. In terms of parity, the findings revealed that many respondents (56.4%) had between 0 and 2 children. Furthermore, most of the participants (71.6%) experienced 3–4 pregnancies.

Data on the knowledge of participants regarding folic acid deficiency are shown in Table 2, whereas findings on awareness are shown in Table 3. Among all the participants, 30.9% accurately recognized that folic acid is a water-soluble vitamin. A quarter of the participants (25.5%) correctly identified the preconception period as the optimal time for women of reproductive age to ensure adequate folic acid levels. Furthermore, a significant majority (79.9%) correctly acknowledged that folic acid deficiency poses a public health concern. Slightly over half of the participants (51.3%) knew that folic acid deficiency can lead to neural tube defects, while 70.9% correctly recognized that folic acid deficiency may cause anaemia. Moreover, most participants (67.5%) correctly reported that unfortified polished rice and noodles/pasta are not reliable sources of naturally occurring folic acid.

**Table 2:** Knowledge of folic acid deficiency among respondents (*N* = 388)

Variable	n (%)
**Folic acid is a water-soluble vitamin**	
Yes	120 (30.9)
No	268 (69.1)
**What is the best time for a woman of reproductive age to be folic acid sufficient?**	
Preconception	99 (25.5)
After conception	289 (74.5)
**Folic acid deficiency is of public health concern**	
Yes	309 (79.6)
No	79 (20.4)
**Deficiency of folic acid may cause neural tube defects**	
Yes	199 (51.3)
No	189 (48.7)
**A deficiency of folic acid may cause anaemia**	
Yes	275 (70.9)
No	113 (29.1)
**Which of the following is not a good source of naturally occurring folic acid?**	
Dark green leafy vegetables	63 (16.2)
Dried beans and peas (legumes)	3 (0.8)
Citrus fruits and juices	60 (15.5)
Unfortified polished rice and noodles/pasta	262 (67.5)

**Table 3:** Awareness and use of folic acid among study participants (*N* = 388)

Variable	n (%)
**Have you ever heard or read about folates/folic acid?**	
Yes	361 (93.0)
No	27 (7.0)
**What is your main source of information regarding folic acid?**	
**Physician**	
Yes	123 (31.7)
No	236 (60.8)
**Midwives/Nurse**	
Yes	253 (65.2)
No	106 (27.3)
**TV/Radio**	
Yes	99 (25.5)
No	260 (67.0)
**Do you know the importance of folic acid?**	
Yes	299 (77.1)
No	89 (22.9)
**Do you know the food sources of folic acid?**	
Yes	294 (75.8)
No	94 (24.2)
**Have you ever taken folic acid supplements or multivitamin**	
Yes	343 (88.4)
No	45 (11.6)
**Are you currently taking any folic acid supplements?**	
Yes	152 (39.2)
No	236 (60.9)

**Fig. 1:**
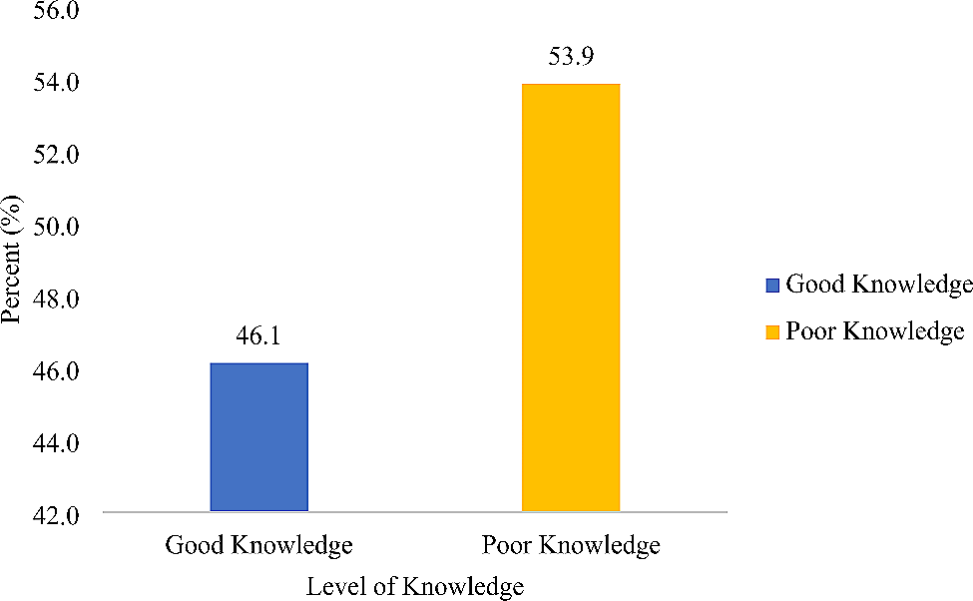
Participants’ FA knowledge level

**Fig. 2:**
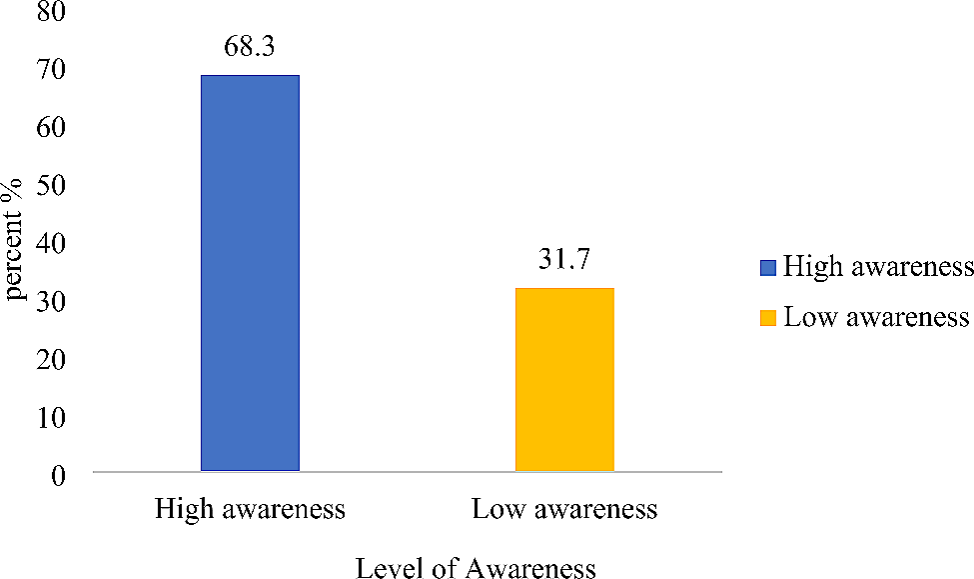
Level of FA awareness among participants

**Fig. 3:**
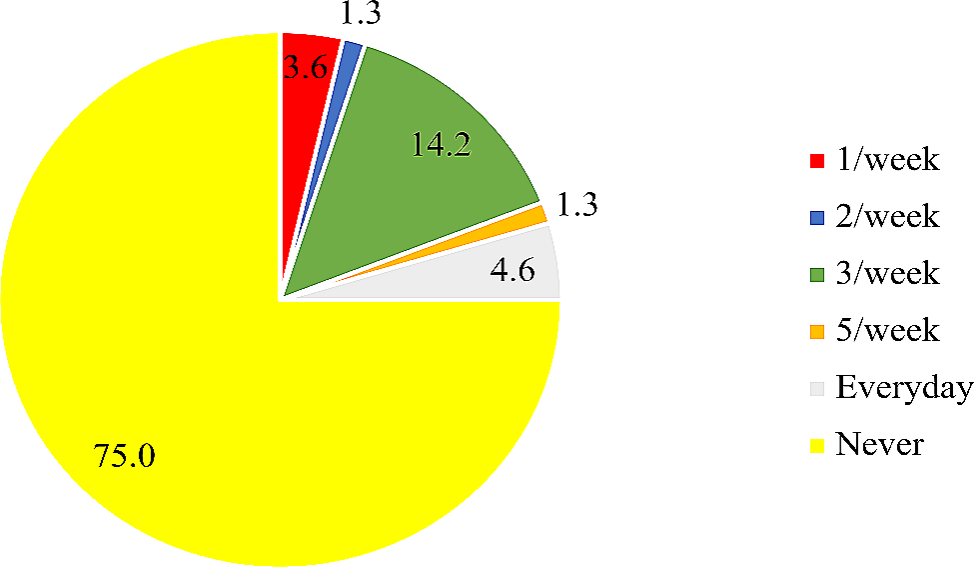
Use of folate supplements among participants

**Fig. 4:**
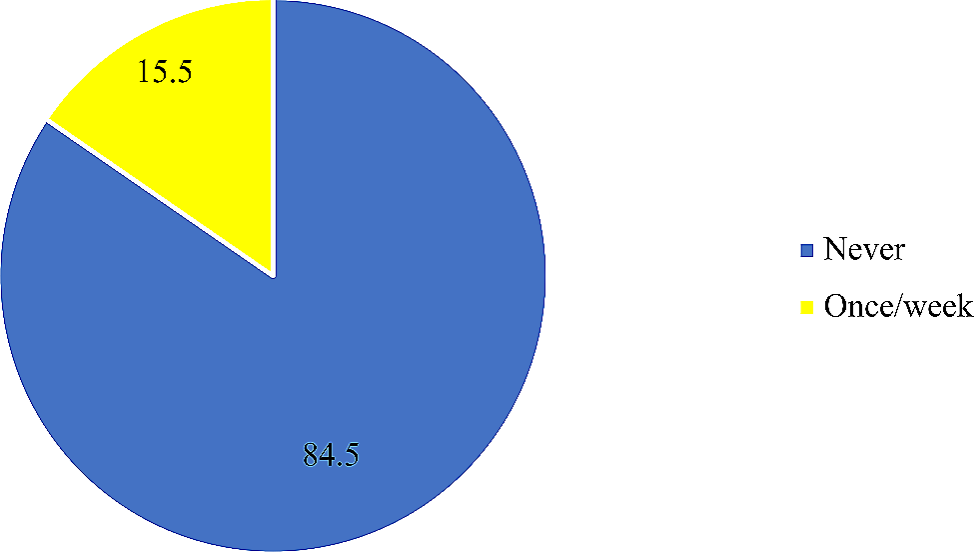
Intake of folic acid-fortified foods

In all, approximately 68% of respondents showed a good level of awareness, while approximately 46.1% of the women who had good knowledge regarding folic acid had a score ≥ 4 out of 6 questions asked (Figs. 1 and 2). The use of folic acid supplements and the intake of folic acid-fortified foods were rather low, with 75% of participants reporting never taking folic acid supplements and nearly 85% having not consumed any folic acid -fortified products in the past week (Figs. 3 and 4). Additionally, awareness and knowledge of folic acid were associated with folic acid supplement use per week, P value < 0.01 (Tables 4 and 5).

**Table 4:** Association between knowledge score and FA use per week

*FA use per week	FA knowledge category		P value
	Good knowledge (n %)	Poor knowledge (n %)	
Never	114 (39.2)	177 (60.8)	**< 0.01**
Once	5 (35.7)	9 (64.3)	
Twice	0 (0.0)	5 (100.0)	
Thrice	55 (100.0)	0 (0.0)	
Quintuple	0 (0.0)	5 (100.0)	
Everyday	5 (27.8)	13 (72.2)	

*FA: Folic acid

**Table 5:** Association between awareness score and FA use per week

*FA use per week	FA awareness category		P value
	High awareness (n %)	Low awareness (n %)	
Never	180 (61.9)	111 (38.1)	**< 0.01**
Once	10 (71.4)	4 (28.6)	
Twice	5 (100.0)	0 (0.0)	
Thrice	55 (100.0)	0 (0.0)	
Quintuple	5 (100.0)	0 (0.0)	
Everyday	10 (55.6)	8 (44.4)	

*FA: Folic acid

## Discussion

Folate/folic acid plays a crucial role in the achievement of healthy pregnancy and lactation. Not only do pregnant and lactating women need to understand the value of folate/ folic acid, but they must also be able to modify and evaluate their diet to support both normal physiological changes of the women for optimal growth and the development of the foetus and offspring [13].

This study revealed that the participants had a good level of high awareness (68.3%), but their knowledge level was significantly lower (46.1%). Compared to previous studies conducted in Australia and China, where 36.5% and 57.0% of women had high awareness of folic acid supplementation, respectively, the current findings were higher [14, 15]. However, the awareness level was lower than that in a study conducted in the USA, which reported that 88.0% of women had high awareness of folic acid [16]. This difference in awareness level could be attributed to the higher percentage of university graduates in the USA study (65.6%) compared to the present study (14.9%).

In our study, it was found that 51.5% of pregnant and lactating women had knowledge regarding the preventive role of folic acid in neural tube defects (NTDs), and 25.5% of the respondents were aware of the recommended timeframe for folic acid supplementation to prevent birth defects. However, these findings were lower compared to a study conducted in China, where 82.7% of pregnant women knew that folic acid prevents NTDs, and 64.5% were aware of the optimal period for ensuring folic acid sufficiency among women of reproductive age [17]. Moreover, our findings were also lower than those of a previous study reporting that over 70% of pregnant women were knowledgeable about the preventive effects of folic acid and the optimal timing for folic acid sufficiency [18]. Apart from the inherent individual variabilities in knowledge and awareness of respondents which were a result of differences in educational background, socio-economic status and educational status/levels among others, the variations in the folate/ folic acid knowledge and awareness across studies could also be mainly attributed to the different cut-offs and testing scales used for the assessment. Some studies used norm-referenced testing which is based on the average performance of the group, others used criterion-referenced testing which uses a predefined mark as good while others did not mention the assessment tool used [13–21]. Even when criterion-referenced testing was used, the cut-off for good knowledge or awareness differed and typically ranged between 70 and 85% and as such makes comparison difficult.

In this study, the overall frequency of daily folic acid intake among the participants was reported to be 4.6%. This finding differs from previous studies conducted in the US and Qatar, where 25.0% and 55.4% of women, respectively, reported taking folic acid supplements daily [16, 19]. The low utilization of folic acid supplements among pregnant and lactating women is concerning, considering that multiple studies have demonstrated the potential of daily folic acid intake in preventing various health issues associated with folic acid deficiency, such as cancer, preterm birth, low birth weight, and NTDs [20]. Some studies attribute the low use of folic acid supplements by women to the adverse effects such as nausea and vomiting [3, 14, 17, 18].

Our study showed a significant association between folic acid use and the level of knowledge and awareness among participants regarding folic acid and its role in human health. In accordance with prior studies [21, 22], it becomes evident that heightened knowledge, suitable guidance, efficient communication, and health enlightenment concerning iron and folic acid supplements (IFAS) amid expectant mothers culminate in remarkable enhancements in their adherence. To achieve such outcomes, it is imperative to disseminate targeted and concentrated information and administer comprehensive instruction and counsel about the significance of IFAS during pregnancy. Conversely, numerous studies have identified challenges in behavioural adjustments as the underlying cause of the discrepancy between folic acid awareness, knowledge, and utilization [23–25]. The use of folic acid supplements necessitates behavioural changes influenced by various factors, such as awareness, knowledge, educational background, and pregnancy planning [26].

The findings of this study emphasize the importance of expanding educational campaigns to promote the use of folic acid and enhance knowledge about its benefits. Several countries have implemented such campaigns with positive outcomes. For instance, in Australia, a national folic acid campaign launched in 1997 resulted in a significant increase in awareness among women of childbearing age. The percentage of women who understood the importance of folic acid in preventing NTDs rose from 25.5% in 1994 to 77.0% in 2007. Similarly, the percentage of women who knew the appropriate timing for folic acid intake increased from 11.5 to 38.9% in 2007, and the usage of folic acid supplements also saw a rise from 37.2% in 1998 to 63.7% in 2007 [21] The United States also conducted national folic acid campaigns since the mid-1990s, leading to consistent increases in awareness. The percentage of women who had heard of folic acid rose from 52.0% in 1995 to 84.0% in 2005, while the proportion of women who understood its role in preventing NTDs increased from 4.0 to 19.0%. Additionally, the use of folic acid supplements increased from 28.0 to 33.0% during the same study period [21].

Additionally, our findings underscore the significant role of midwives and nurses in raising awareness among pregnant and lactating women about the importance of folic acid during these crucial periods. Most participants (65.2%) attributed their knowledge to the efforts of healthcare professionals. However, the roles of television, radio, doctors, and nutritionists were not as prominent. This highlights the potential for healthcare systems to further engage and educate pregnant and lactating mothers regarding folic acid intake during pregnancy and lactation. Similar results have been reported in studies conducted in Saudi Arabia and Nigeria, where health workers emerged as the primary source of information about folic acid for women [22, 27]. Moreover, additional efforts are needed to enhance understanding and awareness of folic acid through television and radio platforms, given the participants’ higher exposure to such media channels.

### Strengths and limitations of the study

This cross-sectional study on folic acid supplementation among pregnant and lactating women in Dodowa, Ghana, had several strengths. The use of a cross-sectional design allowed for a snapshot of knowledge and practices within a specific period, while a descriptive approach provided insights into prevalence and distribution. The study’s focus on a specific geographic area enhanced its relevance and applicability for targeted interventions. The use of standardized data collection tools and techniques ensured consistency and reliability.

However, there were limitations to consider. The cross-sectional design could not establish causal relationships, and self-reported data introduced the possibility of recall and social desirability biases. The sample size and sampling technique may limit generalizability, as the study was conducted in a specific region and may not represent the entire population. Also, the norm-referenced testing used to grade the knowledge and awareness score may be different from the criterion reference test that is used in some studies and as such they may be variations in the levels of good and the self-administered questionnaires were developed based on relevant literature and expert recommendations. Future research with longitudinal designs and larger, representative samples could further enhance the understanding of folic acid awareness, knowledge and use.

## Conclusions

Clearly, the study revealed gaps in knowledge, awareness, and use of folic acid among pregnant and lactating women in the peri-urban community. These findings underscore the need for educational campaigns and interventions to promote folic acid supplementation and fortification involving healthcare providers, media, and public health initiatives. The findings may inform nutrition education plans that will help reduce hidden hunger and improve nutrition security and advise policy actions towards achieving Sustainable Development Goal 2 in Ghana.

## Data Availability

The datasets utilized and analysed in the present study are available from the corresponding author upon reasonable request.

## Abbreviations

Vitamin B_6_: Pyridoxine
Vitamin B_12_: cobalamin
Vitamin B_9_: Folate
DFE: Dietary folate equivalents
DNA: Deoxyribonucleic acid
FA: Folic acid
IFAS: Iron and folic acid supplementation
IRB: Internal Review Board
M: Mean
NTDs: Neural tube defects
RNA: Ribonucleic acid
SD: Standard deviation
USA: United States of America
